# Neural self-representation in autistic women and association with ‘compensatory camouflaging’

**DOI:** 10.1177/1362361318807159

**Published:** 2018-10-24

**Authors:** Meng-Chuan Lai, Michael V Lombardo, Bhismadev Chakrabarti, Amber NV Ruigrok, Edward T Bullmore, John Suckling, Bonnie Auyeung, Francesca Happé, Peter Szatmari, Simon Baron-Cohen

**Affiliations:** 1 University of Toronto, Canada; 2 University of Cambridge, UK; 3 National Taiwan University Hospital, Taiwan; 4 University of Cyprus, Cyprus; 5 University of Reading, UK; 6 Cambridgeshire and Peterborough NHS Foundation Trust, UK; 7 GlaxoSmithKline Research and Development, UK; 8 The University of Edinburgh, UK; 9 King’s College London, UK

**Keywords:** adult, autism, camouflaging, compensation, functional magnetic resonance imaging, gender, heterogeneity, mentalizing, self, sex

## Abstract

Prior work has revealed sex/gender-dependent autistic characteristics across behavioural and neural/biological domains. It remains unclear whether and how neural sex/gender differences are related to behavioural sex/gender differences in autism. Here, we examined whether atypical neural responses during mentalizing and self-representation are sex/gender-dependent in autistic adults and explored whether ‘camouflaging’ (acting as if behaviourally neurotypical) is associated with sex/gender-dependent neural responses. In total, *N* = 119 adults (33 typically developing males, 29 autistic males, 29 typically developing females and 28 autistic females) participated in a task-related functional magnetic resonance imaging paradigm to assess neural activation within right temporo-parietal junction and ventromedial prefrontal cortex during mentalizing and self-representation. Camouflaging in autism was quantified as the discrepancy between extrinsic behaviour in social–interpersonal contexts and intrinsic status. While autistic men showed hypoactive right temporo-parietal junction mentalizing and ventromedial prefrontal cortex self-representation responses compared to typically developing men, such neural responses in autistic women were not different from typically developing women. In autistic women only, increasing camouflaging was associated with heightened ventromedial prefrontal cortex self-representation response. There is a lack of impaired neural self-representation and mentalizing in autistic women compared to typically developing women. Camouflaging is heightened in autistic women and may relate to neural self-representation response. These results reveal brain-behaviour relations that help explain sex/gender-heterogeneity in social brain function in autism.

## Introduction

Autism is a neurodevelopmental condition characterized by early-onset social-communication difficulties alongside heightened stereotyped behaviours, narrow interests, insistence on sameness and idiosyncratic sensory responsivity. The autistic population is highly variable from aetiology to phenotype (Lai et al., 2013; Lombardo et al., 2015). Due to this vast heterogeneity, atypicality in some individuals may not generalize to others with the same diagnosis. It is imperative to go beyond the clinical label of autism to better identify important stratification variables that can meaningfully parse the heterogeneity (Kapur et al., 2012; Loth et al., 2017).

One important stratifier is sex/gender (Lai et al., 2015, 2017a). For many years, a 4–5:1 male:female ratio of autism prevalence has been consistently reported, particularly in clinical samples (Fombonne et al., 2011). However, meta-analyses of epidemiological studies show that with less biased ascertainment, the ratio is around 3:1 (Loomes et al., 2017). There are two important implications from this updated sex/gender ratio. First, autistic females tend to be under-recognized clinically, unless there are co-existing behavioural, emotional, or cognitive difficulties (Duvekot et al., 2017; Dworzynski et al., 2012). Females may present partly different autistic behavioural characteristics from males (Lai et al., 2015, 2018; Mandy, 2017), and gendered socio-cultural contexts may further lead to the under-recognition of autistic characteristics in females (Dean et al., 2017; Kreiser and White, 2014). Second, there is still a clear male-preponderance, even after accounting for biased ascertainment. This indicates that variables and mechanisms associated with sex and gender may be important modulating factors behind the aetiologies and developmental mechanisms of autism (Werling, 2016).

To reduce the under-recognition of autism in females, researchers are starting to clarify the so-called ‘female presentation’ (i.e. behaviours) of autism that are, on average, more frequently expressed in females than in males, especially in individuals without intellectual or severe communication disabilities. ‘Camouflaging’^1^ (defined as acting as behaviourally neurotypical), as a way of coping in social situations, has been proposed to be more common in cognitively able females, especially in those whose autism is not recognized early in life (Attwood, 2007; Bargiela et al., 2016; Wing, 1981).

Qualitatively, camouflaging comprises masking and compensation techniques; these may include suppressing and controlling behaviours associated with autism that were seen as inappropriate in the situation (e.g. reducing repetitive behaviour, ‘stimming’ or responses to sensory over-stimulation), mimicking or ‘performing’ neurotypical peers’ behaviour during social interaction, forcing oneself to maintain eye contact and other non-verbal communication skills (e.g. displaying facial expressions of emotion or interest), or deriving rules/guidelines and scripting conversation with others accordingly to get through small talk or to make social chats more enjoyable for their social partners (Hull et al., 2017).

Quantitatively, camouflaging can be operationalized as the discrepancy between (1) behaviours measured using interaction-based clinical instruments and (2) self-reported autistic characteristics and performance using objective tests of social cognitive ability (Lai et al., 2017b). Using this operationalized definition, we previously found that autistic females show greater camouflaging than age- and IQ-matched autistic males, and that increasing camouflaging is associated with better cognitive control in autistic females (Lai et al., 2017b). Qualitative studies additionally suggest that camouflaging in autistic individuals may be partly different from the ordinary ‘reputation/impression management’ in neurotypical individuals, owing to its extremely effortful and compensatory nature (Bargiela et al., 2016; Hull et al., 2017). Although much of the cognitive and neural bases behind camouflaging/compensation in autism remain unclear (Livingston and Happé, 2017), it has been shown that autistic adolescents (mostly male) who show good socio-interpersonal skills, despite having poor attribution of mental states, have higher IQ and demonstrate better executive function abilities, but also higher anxiety, than those with poor socio-interpersonal skills and similarly poor attribution of mental states (Livingston et al., 2018).

To understand how sex/gender contributes to the heterogeneity of autism, factorial experimental designs with sex/gender and diagnosis as factors should be utilized to examine sex/gender-similarities and/or differences in autism in contrast to same-sex/gender neurotypical (control) populations (Lai et al., 2015). Under this framework, recent neuroimaging studies predominantly examining brain structure and the functional connectome in cognitively able individuals have revealed that several brain characteristics of autism are likely to be *qualitatively* different between males and females, for example, cortical folding and white matter organization associated with the orbitofrontal cortex (Lai et al., 2017a). This means that atypical features of autism present in one sex/gender may not be present in another sex/gender, evidenced by significant diagnosis-by-sex/gender interactions revealed in factorial designs. This implicates potential sex/gender-dependent neurodevelopmental pathways for autism and may further explain behavioural sex/gender differences that underlie the ‘female presentation’ of autism.

Although some neural features of autism appear to be sex/gender-dependent, their association with behavioural-cognitive phenotypes remains unclear. Investigations into sex/gender-dependent brain function are particularly lacking. To date, there are only four published small-scale (largest autistic female sample *N* = 16) task-related functional magnetic resonance imaging (fMRI) studies. Each study probes different tasks but all find sex/gender-dependent functional atypicalities in autism (Beacher et al., 2012; Holt et al., 2014; Kirkovski et al., 2016; Schneider et al., 2013), suggesting that atypical neural function in autism may be sex/gender-dependent across several cognitive domains. Although there are rich theoretical premises and empirical studies suggesting atypical ‘social brain’ function in autism, such as a hypoactive mentalizing network (Happé and Frith, 2014; Pelphrey et al., 2011), the literature on this topic has an overwhelming male bias (Philip et al., 2012). This leaves open the possibility that our understanding of social brain development in autism is ‘over-fitted’ to atypicalities present within males and may not generalize well to females. Furthermore, if social brain function in autism is sex/gender-dependent, it will be informative to examine whether such sex/gender dependency is associated with sex/gender differences in behavioural phenomena such as enhanced camouflaging in females, because camouflaging is a social coping strategy that may involve several compensating/masking mechanisms (e.g. self-monitoring and imitation) (Livingston and Happé, 2017).

Here, we aimed to (1) examine whether atypical neural mentalizing and self-representation responses in autism are sex/gender-dependent and (2) test if enhanced camouflaging is associated with compensatory sex/gender-dependent patterns of social brain function. In typically developing (TD) individuals, right temporo-parietal junction (RTPJ) develops into adulthood with an increasing specialization for mentalizing compared to physical judgements about people (Gweon et al., 2012; Saxe et al., 2009), and ventromedial prefrontal cortex (vMPFC) makes a neural self-other distinction with enhanced responses to self-referential than other-referential processing (Kelley et al., 2002; Moran et al., 2006). We have previously reported that autistic men show reduced vMPFC self-representation and RTPJ mentalizing responses compared to TD men (Lombardo et al., 2010, 2011). Building on these prior findings, in this study, we took a region-of-interest approach and focused on RTPJ and vMPFC to investigate sex/gender dependency of neural processing in autism.

This work is based on newly acquired neuroimaging (task-fMRI) data of TD and autistic females (*N* = 57), matched in age and IQ with the previously published TD and autistic male data (*N* = 62), using the same neuroimaging and behaviour testing paradigms and platforms. Task-fMRI data of males (Lombardo et al., 2010, 2011) and behavioural camouflaging data across sexes/genders (Lai et al., 2017b) have been previously published and serve as the building blocks for this new investigation. Based on literature showing sex/gender dependency of atypical neural features in autism, we predicted that social brain function in autistic females may be different compared with the known hypoactive responses in autistic males. Furthermore, because autistic females may be more likely to invoke compensatory camouflaging strategies/mechanisms than autistic males of similar intellectual abilities (Lai et al., 2017b), it could be that the hypothesized different social brain functioning would be associated with enhanced behavioural camouflaging.

## Methods

### Participants

All participants (*N* = 119) were adult native English speakers with normal/corrected-to-normal vision: 33 TD males, 29 autistic males, 29 TD females and 28 autistic females (Table 1). They all reported cis-gender identity based on a single item inquiring their birth-assigned sex and another on their identified gender. Groups were not statistically different on age or full-scale IQ (FIQ) on the Wechsler Abbreviated Scales of Intelligence (WASI) (Table 1). Exclusion criteria for all participants included a history of or current psychotic disorders, substance-use disorders, severe head injury, genetic disorders associated with autism (e.g. fragile X syndrome and tuberous sclerosis), intellectual disability (i.e. FIQ < 70), or other medical conditions significantly affecting brain function (e.g. epilepsy).

**Table 1.** Descriptive characteristics across all groups. The columns show descriptive statistics for all four groups and indicated statistics from ANOVAs conducted across the groups regarding the effects of Sex/Gender, Diagnosis and Sex/Gender*Diagnosis interaction.

	TD male (*N* = 33)	Autistic male (*N* = 29)	TD female (*N* = 29)	Autistic female (*N* = 28)	Sex/Gender	Diagnosis	Sex/Gender*Diagnosis
	Mean (SD)	Mean (SD)	Mean (SD)	Mean (SD)	F-statistic (*p*-value)	F-statistic (*p*-value)	F-statistic (*p*-value)
Age	27.94 (6.08)	26.59 (7.04)	27.63 (6.40)	28.19 (7.23)	0.24 (0.62)	0.12 (0.73)	0.61 (0.44)
Age range (years)	18–42	18–41	18–45	18–45	–	–	–
VIQ	110.79 (12.03)	112.93 (15.56)	119.45 (9.03)	114.75 (12.29)	5.55 (0.02)	0.20 (0.66)	2.24 (0.14)
VIQ range	71–137	79–136	99–135	76–137	–	–	–
PIQ	118.52 (11.37)	112.31 (16.90)	116.52 (8.36)	110.75 (17.35)	0.49 (0.48)	5.60 (0.02)	0.007 (0.93)
PIQ range	93–135	75–137	96–128	67–137	–	–	–
FIQ	116.27 (11.63)	114.14 (16.42)	120.45 (6.79)	114.46 (13.56)	1.02 (0.31)	2.91 (0.09)	0.70 (0.41)
FIQ range	86–137	75–135	106–133	84–130	–	–	–
ADI-R: Reciprocal-Social-Interaction	–	18.07 (5.07)	–	16.12 (4.75)	2.16 (0.15)	–	–
ADI-R: Communication	–	15.17 (4.24)	–	12.65 (4.34)	4.73 (0.03)	–	–
ADI-R: RRB	–	5.97 (2.76)	–	4.08 (1.79)	8.85 (0.004)	–	–
ADOS: SA (updated algorithm)	–	7.52 (4.63)	–	3.71 (3.60)	11.92 (0.001)	–	–
ADOS: SA + RRB (updated algorithm)	–	9.55 (5.60)	–	4.18 (3.80)	17.82 (9.17e–5)	–	–
ADOS: Communication + Social Total (WPS-published algorithm)	–	7.86 (4.60)	–	3.93 (3.28)	13.72 (4.93e–4)	–	–
AQ	15.24 (6.89)	32.59 (8.20)	11.24 (4.41)	39 (6.19)	0.68 (0.41)	342.89 (2.20e–16)	18.55 (3.51e–5)
RMET correct score	27.27 (3.69)	21.66 (6.29)	28.79 (2.38)	22.43 (6.53)	1.62 (0.21)	42.40 (2.04e–9)	0.17 (0.68)
Camouflaging Score	–	–0.17 (0.38)	–	0.18 (0.33)	13.91 (4.56e–4)	–	–
Mean FD	0.10 (0.06)	0.13 (0.06)	0.12 (0.10)	0.13 (0.06)	0.32 (0.57)	1.79 (0.18)	0.96 (0.33)
Mean DVARS	12.46 (4.11)	13.51 (4.99)	14.58 (9.58)	12.76 (3.70)	0.45 (0.50)	0.08 (0.78)	1.69 (0.20)
Maximal DVARS	39.33 (28.82)	55.21 (44.42)	57.98 (66.21)	47.29 (27.69)	0.53 (0.47)	0.16 (0.69)	2.67 (0.10)

ANOVA: analysis of variance; TD: typically developing; SD: standard deviation; VIQ: verbal IQ; PIQ: performance IQ; FIQ: full-scale IQ; ADI-R: Autism Diagnostic Interview–Revised; ADOS: Autism Diagnostic Observation Schedule; WPS: Western Psychological Services; SA: social affect; RRB: restricted and repetitive behaviour; AQ: Autism Spectrum Quotient; RMET: Reading the Mind in the Eyes Test; FD: frame-wise displacement.

The inclusion criterion for both male and female autistic participants was a formal clinical diagnosis of International Statistical Classification of Diseases and Related Health Problems 10th Revision (ICD-10) childhood autism or Asperger’s syndrome, or *Diagnostic and Statistical Manual of Mental Disorders* (4th ed., text rev.; DSM-IV-TR) autistic disorder or Asperger’s disorder, as assessed by a psychiatrist or clinical psychologist in the National Health Service, UK. Since all participants were adults, we further considered available information of developmental history to include only those with clinically evident childhood autistic symptoms, for example, from information collected using the Autism Diagnostic Interview–Revised (ADI-R) (Lord et al., 1994) where possible, or from the participants’ clinical diagnosis letters shared with the research team to determine eligibility. We used this clinically based criterion for inclusion for the purpose of sampling autistic individuals currently diagnosed by specialists in mental health services in the daily practice and to align with best clinical practice as recommended by the UK National Institute for Health and Clinical Excellence (NICE) guideline (Pilling et al., 2012); see further details in Supplementary Material. For assessing levels of autism characteristics, we administered the Autism Spectrum Quotient (AQ) (Baron-Cohen et al., 2001), module 4 of the Autism Diagnostic Observation Schedule (ADOS) (Lord et al., 2000), and ADI-R (Lord et al., 1994) where possible, before the fMRI session. Autistic male and female groups were not different on ADI-R Reciprocal-Social-Interaction scores or Reading the Mind in the Eyes Test (RMET) performance (Table 1). Participants’ informed consent was obtained in accord with procedures approved by the Suffolk Local Research Ethics Committee.

### fMRI task design and data acquisition

This was a 2 × 2 within-subjects factorial block design where participants were asked to make either reflective ‘Mentalizing’ or ‘Physical’ judgements about two target individuals: the ‘Self’ or a familiar non-close ‘Other’ (the British Queen) (Lombardo et al., 2010, 2011). For self-mentalizing (SM) blocks, participants judged on a scale from 1 to 4 (where 1 = ‘not at all likely’ and 4 = ‘very likely’) how likely they themselves would be to agree with opinion questions that focused on mental characteristics (e.g. ‘How likely are you to think that keeping a diary is important?’). On other-mentalizing (OM) blocks, the same mentalizing judgements were made, except this time, in reference to how likely the British Queen would be to agree with the opinion questions (e.g. ‘How likely is the Queen to think that keeping a diary is important?’). During self-physical (SP) blocks, participants judged how likely they would be to have specific physical characteristics (e.g. ‘How likely are you to have bony elbows?’). Conversely, the same physical judgements were made during other-physical (OP) blocks, except that participants rated these questions with the Queen as the target person (e.g. ‘How likely is the Queen to have bony elbows?’). This fMRI task was designed in a way that there was no correct or incorrect answer indicating objective social cognitive performance behaviourally. All opinion questions were acquired from Jason Mitchell’s lab and have been used in previous studies on reflective mentalizing judgements of the self and others that reliably elicit robust and consistent activity in mentalizing and self-referential neural circuits (Jenkins et al., 2008; Mitchell et al., 2006). Stimuli did not differ per condition in the number of characters, syllables, frequency or valence.

All participants completed one scanning session with one fMRI run. Within this run, there were 20 trials within each condition and five blocks per condition. Each trial type was presented in blocks of four trials and the trial-duration was 4 s each (16 s per block). After each block, there was a rest period of 16 s where participants fixated on a cross on the screen. All trials within blocks and all blocks throughout the functional run were presented in pseudorandom order. Stimulus presentation was implemented with DMDX software on a computer synchronized with the onset of the functional run to ensure accuracy of event timing.

Imaging was performed on a 3T GE Signa Scanner at the Cambridge Magnetic Resonance Imaging and Spectroscopy Unit. The fMRI run consisted of 325 whole-brain T2*-weighted echoplanar images (slice thickness, 3 mm; 0.8 mm skip; 33 axial slices; repetition time, 2000 ms; echo time, 30 ms; flip-angle, 90°; matrix, 64 × 64; field-of-view, 240 mm, sequential slice acquisition). The first five time-points were discarded to allow for T2-stabilization. A high-resolution spoiled gradient anatomical image was acquired for each subject for registration purposes.

### fMRI data analysis

fMRI data pre-processing and individual-subject generalized linear model (GLM) analyses were implemented using SPM5 (http://www.fil.ion.ucl.ac.uk/spm). The pre-processing steps included slice-timing correction, realignment to the mean functional image, co-registration of the functional images with structural image, segmentation of the structural image, normalization into standard MNI space by applying the transformations estimated from segmentation and spatial smoothing with an 8-mm full-width-half-maximum Gaussian kernel. Data were evaluated for in-scanner motion effects across groups using mean frame-wise displacement (FD), mean DVARS and maximal DVARS (Power et al., 2012); there were neither significant diagnosis-by-sex/gender interactions for any of them nor any main effects of Sex/Gender or diagnosis (Table 1). Descriptively, head motion was minimal with all groups having mean FD < 0.14 mm. The FD range was 0–4.19 mm across all subjects. No group differences exist on minimal FD or maximal FD (all p > 0.104).

Individual-subject general linear modelling in SPM used a canonical hemodynamic response function convolved to each trial. High-pass temporal filtering with a cut-off of 128 s was applied to remove low-frequency drift, and global changes were removed by proportional linear scaling. Serial autocorrelations were estimated with a restricted maximum likelihood algorithm with an autoregressive model of order 1. Second-level group analyses were conducted with region-of-interest (ROI) analyses, implemented within R (https://www.r-project.org). Because we focused on vMPFC and RTPJ responses congruent with past work (Lombardo et al., 2010, 2011), we used the same meta-analytically defined independent ROIs from these past papers, which included studies on both male and female individuals. These ROIs, therefore, could be considered sex/gender-common brain regions associated with self-representation and mentalizing. Percent-signal-change was computed for all voxels then averaged within the ROI. We then computed contrast values for the two main effects of interest: ‘Self > Other’ and ‘Mentalizing > Physical’. These contrast values were assessed for the predicted diagnosis-by-sex/gender interactions with a linear model including Sex/Gender, Diagnosis, Diagnosis*Sex/Gender and additional nuisance covariates of age and FIQ. Age and FIQ were included because the ranges were substantial and we wanted to guard against this confounding variability. Significant diagnosis-by-sex/gender interactions were evaluated based on a conventional alpha-level of 0.05.

### Behavioural index of camouflaging

As camouflaging could be defined as (consciously or unconsciously) compensating for and/or masking difficulties in social–interpersonal situations, we used the same operationalized definition from past work (Lai et al., 2017b): the discrepancy between extrinsic behavioural presentation in social–interpersonal contexts and the person’s intrinsic status. We used both the AQ score and RMET correct score as reflecting intrinsic status (i.e. self-rated dispositional traits and performance-based socio-cognitive/mentalizing capability). We used the updated algorithm Social Affect (SA) domain score of the ADOS module 4 (Hus and Lord, 2014) to reflect extrinsic presentation. The three scores were first standardized (S_ADOS_, S_AQ_ and S_RMET_) within the present sample of autistic men and women by mean-centring (to the whole autism sample in this study) and scaling (i.e. divided by the maximum possible score of each) to generate uniformly scaled measures that can be arithmetically manipulated. The first estimate of camouflaging was quantified as the difference between self-rated autistic traits and extrinsic behaviours (CF1 = S_AQ_ − S_ADOS_), and the second estimate between mentalizing ability and extrinsic behaviours (CF2 = −S_RMET_ − S_ADOS_). Finally, using principal component analysis, the first principal component score of CF1 and CF2 (accounting for 88% of the variance) was taken as a single, parsimonious measure of camouflaging for all further analyses. This measure should be interpreted by relative values (i.e. higher scores indicate more camouflaging) rather than absolute values (e.g. a score of 0 simply means it is smaller than 1 and bigger than -1, but does not indicate ‘no camouflaging’). This operationalization only allows for estimating camouflaging in autistic individuals, as it partly derives from the ADOS score which was not available in TD participants. It is our view that this approach remains informative, as qualitative studies have suggested that camouflaging in autism may be partly different from similar phenomenon in neurotypical individuals (Bargiela et al., 2016; Hull et al., 2017). Finally, all analyses related to the camouflaging index were repeated to check consistency of findings using two other different versions of ADOS score: the Western Psychological Services, WPS-published ‘diagnostic algorithm’ Communication + Social Interaction Total score (Lord et al., 2000) as per our previous publication (Lai et al., 2017b), and a broader conceptualization of camouflaging using the updated algorithm SA + Restricted and Repetitive Behaviour (RRB) score (Hus and Lord, 2014).

### Neural activation–camouflaging relationship analysis

We assessed the relationship between neural activation and camouflaging scores with partial correlations computed with *robust regression*, a form of regression that is robust to the impact of bivariate outliers (Wager et al., 2005), and co-varying for age and FIQ, separately for autistic males and females. We further tested for a difference in the strength of activation–camouflaging correlations between the sexes/genders. This was achieved by converting correlations to *z*-scores with Fisher’s *r*-to-*z* transform and then finding the *z* statistic for the difference between the two independent correlations, using the *paired.r* function within the *psych* library in R.

## Results

### fMRI task behavioural results

Repeated measures analysis of variance (ANOVA) with mean reaction time (RT) during the fMRI task as the dependent variable and two between-subjects factors (Sex/Gender and Diagnosis) and two within-subjects factors (Target (Self and Other) and Judgement (Mentalizing and Physical)) revealed no significant four-way or three-way interactions (*F* < 2, *p* > 0.15) including between-subjects factors of Sex/Gender and Diagnosis, and no two-way Sex/Gender*Diagnosis interaction (*F*(1, 115) = 1.03, *p* = 0.31). There was a main effect of Sex/Gender (*F*(1, 115) = 9.54, *p* = 0.003) driven by faster RT in females than males. Thus, the evidence here suggests that there is no clear pattern of behavioural difference in how the four groups perform the task, other than females as a whole responding faster than males.

**Figure 1.**
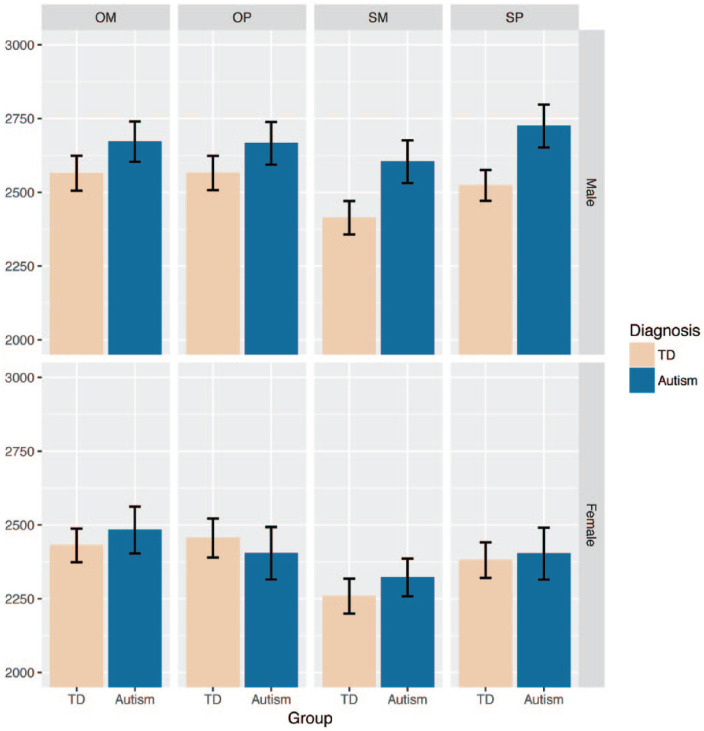
fMRI task behavioural data (mean reaction time, RT). SM: self-mentalizing: SP: self-physical; OM: other-mentalizing; OP: other-physical. Bars depict the mean. Error bars indicate ± 1 standard error of the mean.

All other effects reaching significance have been reported previously in a paper on just the males from this data set (Lombardo et al., 2010) and therefore do not represent novel findings. For example, among two-way interactions, there was a Target*Diagnosis interaction (*F*(1, 115) = 5.25, *p* = 0.02) driven by faster RT for Self compared to Other in the TD group, but little of this effect in the autism group. Among the within-subjects factors, there was also a general Target*Judgement interaction (*F*(1, 115) = 27.83, *p* = 6.31e–7) which was driven primarily by faster mean RT during the SM condition compared to all other conditions. Among main effects, there was a main effect of Judgement (*F*(1, 115) = 12.96, *p* = 0.0005; faster RT for Mentalizing), Target (*F*(1, 115) = 26.84, *p* = 9.55e–07; faster RT for Self), but no main effect of Diagnosis (*F*(1, 115) = 1.96, *p* = 0.16). See Figure 1 and Table 2 for summary of fMRI task mean RT data.

**Table 2.** Summary descriptive statistics for fMRI task behavioural data (mean reaction time, RT).

	TD male	Autistic male	TD female	Autistic female
SM	2413.65 (325.83)	2603.76 (389.61)	2258.86 (316.61)	2322.34 (338.24)
SP	2523.14 (300.49)	2724.70 (392.13)	2380.90 (324.18)	2403.01 (464.82)
OM	2564.49 (338.17)	2671.83 (366.28)	2430.40 (306.97)	2482.90 (418.81)
OP	2565.45 (331.05)	2665.93 (388.93)	2455.63 (355.09)	2404.06 (470.09)

TD: typically developing; SM: self-mentalizing; SP: self-physical; OM: other-mentalizing; OP: other-physical.

Each cell shows the mean and standard deviation (in parenthesis) for each group and condition.

In short, analysis of behavioural data from the fMRI task indicated no significant interactions including between-subjects factors of Sex/Gender or Diagnosis, indicating that the behavioural patterns of performance were similar across autistic men and women in our sample.

### Neural diagnosis-by-sex/gender interactions

**Figure 2.**
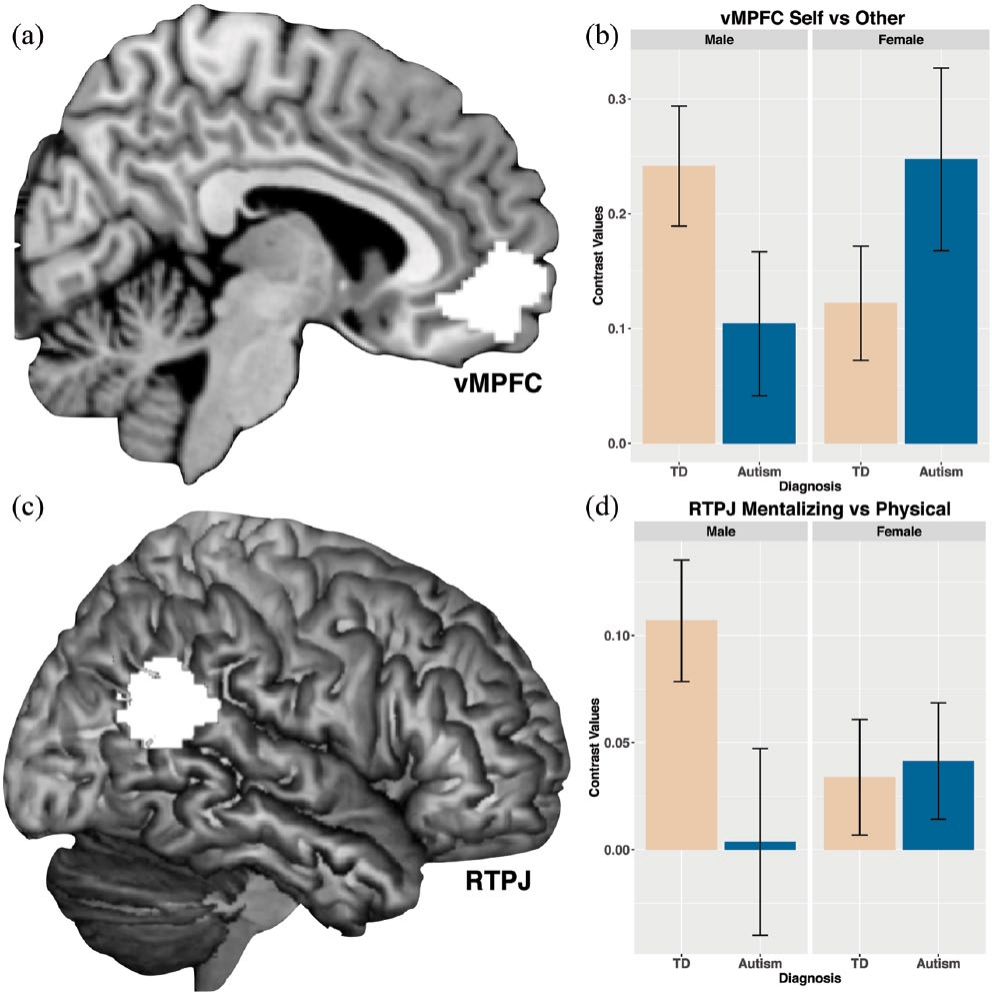
Diagnosis-by-sex/gender interaction effects for vMPFC Self > Other activation (a and b) and RTPJ Mentalizing > Physical activation (c and d). Higher values on the y-axis indicate greater activation. Bars depict the mean. Error bars indicate ± 1 standard error of the mean.

At the level of neural responses, we tested for the predicted diagnosis-by-sex/gender interactions in vMPFC and RTPJ activation. For the Self > Other contrast, vMPFC showed the predicted diagnosis-by-sex/gender interaction (*F*(1, 113) = 4.89, *p* = 0.029, partial η2 = 0.047). This interaction was driven by evident hypoactivation of vMPFC in autistic males compared to TD males (Cohen’s *d* = 0.44), but a nominal effect in the opposite direction for autistic females versus TD females (Cohen’s *d* = −0.38) (Figure 2(a) and (b)). The RTPJ Mentalizing > Physical contrast also showed a significant diagnosis-by-sex/gender interaction (*F*(1, 113) = 4.11, *p* = 0.045, partial η^2^ = 0.035). Descriptively, this interaction was driven by an evident TD > Autism effect in males (Cohen’s *d* = 0.52), but a nominal effect in the opposite direction for females (Cohen’s *d* = −0.20) (Figure 2(c) and (d)).

### Sex/gender-specific vMPFC activation–camouflaging relationship

As reported in our previous work using a largely overlapping sample (Lai et al., 2017b), autistic women, on average, scored higher on camouflaging (calculated using the ADOS updated algorithm SA domain score) compared with autistic men, indicating enhanced camouflaging (*F*(1, 55) = 13.91, *p* = 4.56e–4, Cohen’s *d* = 0.99). Camouflaging was not significantly correlated with age, VIQ, or PIQ in either group.

**Figure 3.**
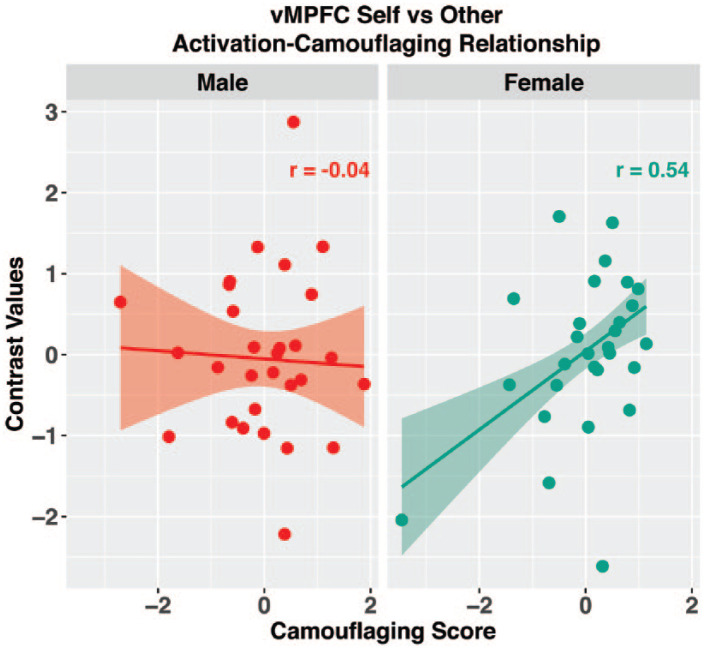
Activation–camouflaging relations for the Self > Other contrast at vMPFC. Plotted activation (contrast values) and camouflaging scores (calculated via using the ADOS updated algorithm SA domain score) are standardized within each sex/gender. Correlations are computed with a robust regression model to be insensitive to outliers and co-varies for age and FIQ. The plotted best fit line and 95% confidence band are fit using robust regression.

Confirming our prediction, there was a significant positive correlation between vMPFC Self > Other activation and camouflaging scores in autistic females (*r* = 0.54, *p* = 0.019), but there was no significant association in autistic males (*r* =−0.04, *p* = 0.86). The difference between the two correlations was significant (*z* = 2.3, *p* = 0.02) (Figure 3). In contrast to vMPFC, when considering RTPJ Mentalizing > Physical activation, there was no significant correlation present in either females (*r* = 0.19, *p* = 0.41) or males (*r* = 0.15, *p* = 0.55), and no significant difference between the correlations (*z* = 0.15, *p* = 0.88).

All these findings hold when camouflaging was calculated via using the ADOS updated algorithm SA + RRB score or the WPS-published ‘diagnostic algorithm’ score; see Supplementary Material for details.

## Discussion

We examined whether atypical neural self-representation and mentalizing responses within vMPFC and RTPJ are sex/gender-dependent in autism and whether camouflaging, as we provisionally operationalized, is associated with such social brain function. We identified sex/gender-dependent neural activation patterns: whereas autistic men showed reduced vMPFC self-representation and RTPJ mentalizing responses compared with TD men, autistic women showed no significant differences from TD women (although, descriptively, with a small effect in the opposite direction). In addition, enhanced camouflaging in autistic women, but not men, was related to greater vMPFC neural self-representation response; such findings remained the same irrespective of the version of ADOS algorithm scores used for estimating camouflaging. We do not consider the nominal mean differences of PIQ and VIQ across diagnostic and sex groups to be confounds since neither was significantly correlated with the outcome measures of camouflaging or level of neural activation, and variance associated with IQ was accounted for in the statistical models. In brief, the present findings highlight plausible sex/gender dependency in social brain function in autism and point to a link between neural self-representation and a social coping phenomenon, camouflaging, that is heightened in autistic women.

Sex/gender dependency in aspects of atypical brain structure in autism has been noted across several studies (Lai et al., 2017a). Sex/gender dependency in brain function has also been reported in small-scale task-fMRI studies examining empathy, emotion recognition, spontaneous mental-state attribution (Holt et al., 2014; Kirkovski et al., 2016; Schneider et al., 2013) and mental rotation (Beacher et al., 2012). With a sample twice as large as these previous task-fMRI studies, we found sex/gender dependency in the domains of self-referential cognition and, to a lesser extent, mentalizing. Such evidence supporting aspects of qualitative sex/gender differences in the autistic brain implies that it is important to consider sex/gender-differential effects as they may play considerable modulating roles and alter the interpretations of past literature (Lai et al., 2017a). For example, a key caveat of claiming ‘atypical (hypoactive) social brain function’ in autism (Dichter, 2012; Pelphrey et al., 2011) is that the inferences are largely drawn from heavily male-biased samples (Di Martino et al., 2009; Philip et al., 2012). Actually, the atypicality, if any, may be quite different in autistic women versus men.

Whether social brain function in autism develops in a sex/gender-differential manner from early in life has yet to be tested. Longitudinal work from infancy will be the key to answering this question (Johnson, 2017; Lombardo et al., 2015; Szatmari et al., 2016). The observed sex/gender differences in autistic social brain functioning could be the product of sex-related biological factors inherent in biology from or before birth, and/or gender-related experiences after birth. Given the notion from past work that brain activation patterns in autism can be affected by simple behavioural manipulations/instructions (Wang et al., 2007), it could be that the socio-cultural environment has gender-differential impacts and may influence social brain function over the lifespan. The socio-cultural environment for autistic females may, on average, impose higher demands relative to males for social-communication and interaction, owing to the typically gendered behaviour and role expectations (Kreiser and White, 2014). Because autistic females (especially those showing mild characteristics earlier in life) may be more pressed to modify their behaviours for camouflaging/compensation (Attwood, 2007; Bargiela et al., 2016; Livingston and Happé, 2017) based on gendered expectations (Kanfiszer et al., 2017; Kreiser and White, 2014; Lai et al., 2015), such influence may engage different experience-dependent mechanisms in the brain that may explain ‘normal’ levels of vMPFC self-representation and RTPJ mentalizing responses.

How autistic individuals camouflage or compensate for their difficulties as they get older, via genetically driven or experience-dependent mechanisms, is an important research horizon that we know very little about. Two types of compensation may point towards different mechanisms (Livingston and Happé, 2017). ‘Shallow compensation’ refers to superficial, inflexible and fragile means for navigating the complex social world (e.g. through use of behavioural rules). An example of this might be ‘keep talking’, which keeps one connected to a listener but may not be sensitive to the context and may result in excessive faux pas (Baron-Cohen et al., 1999; Thiebaut et al., 2016). ‘Deep compensation’ is more flexible, sophisticated and adaptable (e.g. through attribution of mental states, albeit via non-typical routes). Deep compensation may thus be reflected in neural activation patterns distinct from those used for shallow compensation (Livingston and Happé, 2017).

Hence, one plausible interpretation for the present findings is that autistic women, on average, may engage more ‘deep compensation’ than autistic men. In this respect, the fact that the association with camouflaging was found only with vMPFC and not RTPJ is also noteworthy. Speculatively, this may suggest that for autistic women, compensation via enhanced self-reflection is more critical than mentalizing. To camouflage successfully, autistic women may engage substantial insight about their own behaviours in interpersonal and social contexts – specifically, how their behaviours impact others, gauging and managing the impressions they make on others, updating the differences between their natural and camouflaged behaviours, and how such behaviours will achieve the desired goal of being perceived as neurotypical (Hull et al., 2017). A main ingredient in these processes is an ability to represent similarities and differences between oneself and others – a function in which vMPFC plays critical roles (Kelley et al., 2002; Mitchell et al., 2006; Moran et al., 2006; Nicolle et al., 2012). While this link between vMPFC neural self-representation and camouflaging is an intriguing first step, the results do not lend themselves to causal statements. We cannot conclude that a normal level of vMPFC neural self-representation leads to enhanced camouflaging or that enhanced camouflaging leads to normalization of vMPFC neural responses. Disentangling this relationship (through longitudinal or intervention studies) will be an important translational goal. Finally, the present findings do not exclude the possibility that camouflaging in autistic women is associated with other cognitive functions that also involve vMPFC (e.g. emotion regulation, reward processing and decision-making) or beyond. Our fMRI task does not tackle these domains, so no inference can be made about these aspects accordingly.

Overall, our findings are particularly relevant to the bigger issue of heterogeneity in autism. Sex, gender and related variables may form important dimensions for stratifying neuropsychiatric conditions with sex/gender-biases (Joel and McCarthy, 2017), including autism (Lai et al., 2015). Through stratification by sex- and gender-related variables, the deeper translational research goal will be to understand better how specific aetiological and developmental mechanisms of autism diverge among sexes/genders, and contribute to the sex/gender-differential vulnerability (Lai et al., 2015, 2017a; Werling, 2016). Many important empirical questions arise: What drives these diagnosis-by-sex/gender interactions in social brain function and differences in propensity to engage in camouflaging? Are such mechanisms rooted in biological or experiential mechanisms, or their interplay? Are such mechanisms divergent among the sexes and genders across the lifespan? Clarifying these ‘nature versus nurture’ mechanisms associated with sex and gender is key to tracing back the sources of heterogeneity, leading to novel sex- and gender-informed identification and support for autism.

There are important limitations to be considered. First, although the sample size is much larger than all existing task-fMRI studies on the topic (the largest sample reported to date as far as we know), it is still modest and likely under-powered to detect small effect sizes. This means that the lack of group differences in neural activation reported here cannot be unambiguously taken as evidence for claiming the same or ‘intact’ patterns of neural activation during self-representation and mentalizing in women with versus without autism. Much larger samples are needed to validate these findings. Related to sample size is the issue that other aspects of heterogeneity could be present and have important effects on sample variation. Most task-fMRI studies of autism, including ours, are on verbal and intellectually able youth or adults (Jack and Pelphrey, 2017). Whether similarly atypical social brain functions are seen in younger, minimally verbal or intellectually disabled individuals, and whether a sex/gender-dependent pattern is also present in these subgroups, remain unclear until more studies are done using paradigms better tailored to their special characteristics (e.g. passive viewing). Similarly, it is still an open question with regard to how cultural, ethnic, linguistic and socio-economic heterogeneity may impact the present findings.

Second, the moderate sample size also limits our ability to take a hypothesis-free, discovery approach, since statistical power declines with more statistical comparisons. Considering this issue and given our already a priori goals motivated by earlier work on males, we took a conservative approach to focus on two regions most relevant to mentalizing and self-referential processing. One may question whether the findings of no differences between autistic and TD women stem from any normative sex/gender differences in regional specialization of social processing. Nevertheless, the two ROIs we examined (RTPJ and vMPFC) were derived from meta-analyses that included *both* male and female individuals. In addition, there is a lack of evidence so far demonstrating sex/gender-specific ‘social brain’ organization in the human neuroscience literature (Pavlova, 2017). Finally, it is still possible that autistic women utilize different brain regions for mentalizing and self-referential cognition compared with TD women, therefore differences from TD women cannot be revealed by solely examining RTPJ and vMPFC. A hypothesis-free, discovery approach is needed to explore this, yet our data set is not well-powered for this sort of investigation. Future research using much larger samples is required to answer this question.

Third, investigating sex/gender differences in autism is inevitably complicated by the still-unresolved challenges related to ascertainment and clinical measurement. Interpretation of findings depends on study-specific sample ascertainment and characteristics (see Lai et al. (2015) for detailed discussion). Our inclusion rationale aimed at sampling autistic adults identified by current standard clinical practice in the United Kingdom. The study was not designed to match males and females with autism on their scores of ADOS or ADI-R, but primarily matching them on age and IQ. In fact, our study was motivated by recent reports that cognitively able females with autism may appear to be less affected on these ‘gold standard’ diagnostic instruments (Hiller et al., 2014; Lai et al., 2011; Langmann et al., 2017; Rynkiewicz et al., 2016). It may be that measured ‘classical’ autism characteristics and compensatory mechanisms are intertwined in an important way; for example, less-severe ADOS scores could be a result of autistic women engaging in compensation strategies, hiding their atypical social-communication features in an observational setting like the ADOS (Lai et al., 2011; Langmann et al., 2017; Livingston and Happé, 2017; Rynkiewicz et al., 2016). It is still unknown whether a similar pattern of findings would be replicated in female individuals who may be considered more ‘classically autistic’ or ‘severe’ (e.g. being diagnosed early in life or with high ADOS scores).

Fourth, the age-range examined (18–45 years) is quite broad. It will be important to follow up with targeted studies at different life stages, considering the plausible roles of experiential effects and sex/gender-related plasticity in social brain and social-communication development. Specifically, as we do not have data of the age of autism diagnosis, previous exposure to autism-related intervention/support and the extent of social experiences, it is difficult to infer how these experiential factors are causally linked to the present findings. Variations of the opportunities of social experiences and developing social coping mechanisms are likely associated with behavioural and neural compensation in autistic people. Prospective studies on compensation and camouflaging in autism will benefit from better quantification of such experiential factors.

Fifth, as a common issue in studies involving human adults, it is important to acknowledge that processes related to both sex and gender intertwine throughout development, therefore in many scenarios it is difficult to delineate effects of sex from those of gender. Furthermore, we did not measure the different factors underlying sex and gender, respectively (Joel and McCarthy, 2017), that can be used to explore their unique impacts. Future studies would benefit from more comprehensive characterization of the multiple components of sex and gender, and an environmentally and developmentally sensitive lens, to disentangle their respective contributions.

Finally, although camouflaging has been described in the clinical and autobiographical literature of autism for some time (Lai et al., 2017b), it is still a relatively new construct in empirical research. It remains unclear the extent to which camouflaging is associated with more well-established psychological constructs (e.g. imitation, introspection and social anxiety) or similar phenomena that have been described in neurotypical individuals (e.g. impression management, or ‘performance’ as described by the sociologist Erving Goffman). The way we operationalize camouflaging (only in autistic individuals), as well as the efforts to identify neural correlates, should still be considered exploratory. More refined measurements for camouflaging and associated latent constructs are still being developed (Dean et al., 2017; Hull et al., 2017; Lai et al., 2017b; Livingston et al., 2018; Livingston and Happé, 2017; Parish-Morris et al., 2017; Rynkiewicz et al., 2016), and more studies are required to examine the extent to which camouflaging in autism is like that in non-autistic individuals. We consider this work being part of this effort towards obtaining more clarity about what ingredients are involved.

In conclusion, inferences about social brain function in autism appear to depend on sex/gender. Whereas intellectually able autistic men showed reduced vMPFC self-representation and RTPJ mentalizing responses compared to TD men, intellectually able autistic women showed a lack of differences in neural responses compared to TD women. Heightened vMPFC self-representation responses were associated with enhanced camouflaging, but only in autistic women. These insights may lead to new investigations into how sex/gender-related heterogeneity is linked to compensatory mechanisms in autism and provide translational potential for developing novel support of social coping for autistic individuals.

## Supplemental Material

aut-18-0226-File006 – Supplemental material for Neural self-representation in autistic women and association with ‘compensatory camouflaging’ Supplemental material, aut-18-0226-File006 for Neural self-representation in autistic women and association with ‘compensatory camouflaging’ by Meng-Chuan Lai, Michael V Lombardo, Bhismadev Chakrabarti, Amber NV Ruigrok, Edward T Bullmore, John Suckling, Bonnie Auyeung, Francesca Happé, Peter Szatmari and Simon Baron-Cohen in Autism

## Notes

1. We should add that the term ‘camouflaging’ is relatively new, and in exploring this phenomenon, our intention is not to ‘reveal’ or ‘out’ autistic people who are otherwise undiagnosed or ‘hidden’, but rather to understand the effortful strategies that some diagnosed autistic people adopt, sometimes at the cost of their own mental health because such strategies leave them feeling that they are not able to ‘be themselves’; see (Cage et al., 2018; Hull et al., 2017). Camouflaging, therefore, has important implications for mental wellbeing in autism. Equally, the term ‘compensatory’ is widely used in the field of neuropsychology to refer to how an individual adopts strategies to bypass cognitive deficits. The construct is also being investigated in the psychological studies of autism (Livingston et al., 2018; Livingston and Happé, 2017). In this study, we do not use the term ‘compensatory’ with any implication that autistic behaviour is in some way ‘lesser’ or inferior to neurotypical behaviour, but regard autistic behaviour as different, as understood by the neurodiversity framework (Silberman, 2015). We hope autistic people can help us refine the new terminology in this field.
