# Supplementary material for: Neural self-representation in autistic women and association with ‘compensatory camouflaging’
Source: Autism. 2018 Oct 24;23(5):1210–23. doi: 10.1177/1362361318807159 (PMC6589917; doi:10.1177/1362361318807159)
Supplement: aut-18-0226-File006 – Supplemental material for Neural self-representation in autistic women and association with ‘compensatory camouflaging’ [file aut-18-0226-File006.pdf]

Supplementary Material

<u>Methods:</u> Participants

Based on our inclusion rationale of ascertaining clinically diagnosed autistic individuals, we acknowledged that the participants might not all score above the conventional research thresholds on the ADI-R and ADOS ‘diagnostic algorithm’. The same scenario has also been shown in the latest large-scale autism research in Europe, in which the ascertainment attempts to capture the wide heterogeneity of the autism spectrum (Charman et al., 2017). In our sample, all but 2 autistic males reached the diagnostic algorithm cut-offs of ‘autism’ on the ADI-R (which corresponded to the DSM-IV ‘autistic disorder’), with these 2 males being one point below the cut-off on the restricted/repetitive behaviour (RRB) domain. Two autistic females were included but ADI-R was unavailable due to childhood caregivers being unable to be interviewed: one scored above the cut-off for ADOS ‘autism spectrum’ and the other was positive for a diagnosis on the Adult Asperger Assessment (AAA) which incorporates caregiver reports of childhood behaviours (Baron-Cohen, Wheelwright, Robinson, & Woodbury-Smith, 2005). Two other autistic females were included who fell short of the ADI-R diagnostic algorithm thresholds in up to two domains by 1-3 points, but both have a confirmed ICD-10 diagnosis of Asperger’s syndrome by expert clinicians, with evident and clinically significant childhood autistic symptoms documented in their diagnosis letters. The rationale of this inclusion strategy was to allow for possible under-estimation of early developmentally atypical behaviours in caregivers’ recall on the ADI-R, and also to account for RRB symptoms now considered central in DSM-5 (e.g., adherence to routine, idiosyncratic sensory responses) but *not* included in the DSM-IV-based ‘diagnostic algorithm’ of ADI-R.

In terms of socio-demographic characteristics, our sample was composed of long-term UK residents with English as their first language (except for one autistic woman who also spoke another native language), Caucasian by descent (with one neurotypical man and one autistic woman with mixed Caucasian and other ethnic background). We did not measure other socio-economic status.

Behavioural-cognitive and neuroanatomical characterizations including and beyond this sample have been reported in previous MRC-AIMS studies (Lai et al., 2011; Lai et al., 2017; Lai et al., 2012; Lai et al., 2013; Zeestraten et al., 2017), along with detailed project and recruitment information. The sample included here overlaps with the autism groups reported in a previous structural MRI study (Lai et al., 2013) and a behavioural study operationalizing camouflaging (Lai et al., 2017); the male data are the same from published task-fMRI studies (Lombardo, Chakrabarti, Bullmore, & Baron-Cohen, 2011; Lombardo et al., 2010). However, task-fMRI data of females constitute new data not reported in any prior publication and are the focus of the present paper.

<u>Results:</u> Sex/Gender-Specific vMPFC Activation-Camouflaging Relationship (full results with camouflaging calculated via using 3 different versions of ADOS score)

As reported in our previous work using a largely overlapping sample (Lai et al., 2017), autistic women on-average scored higher on camouflaging (calculated using the ADOS updated algorithm SA domain score) compared with autistic men, indicating enhanced camouflaging ($F(1,55)$=13.91, p=4.56e-4, *Cohen's d*=0.99); the pattern was consistent when camouflaging was calculated via using the ADOS updated algorithm SA+RRB score ($F(1,55)$=18.76, p=6.34e-5, *Cohen's d*=1.15) or the WPS-published algorithm score ($F(1,55)$=14.72, p=3.23e-4, *Cohen's d*=1.01). Camouflaging was not significantly correlated with age, VIQ or PIQ in either group.

Confirming our prediction, there was a significant positive correlation between vMPFC Self>Other activation and camouflaging scores in autistic females (r=0.54, p=0.019; findings were consistent when using ADOS updated algorithm SA+RRB score, r=0.50, p=0.026, or WPS-published algorithm score, r=0.48, p=0.03), but there was no significant association in autistic males (r=-0.04, p=0.86; findings were consistent when using ADOS updated algorithm SA+RRB score, r=-0.04, p=0.87, or WPS-published algorithm score, r=-0.10, p=0.64). The difference between the two correlations was significant (z=2.3, p=0.02; findings were consistent when using ADOS updated algorithm SA+RRB score, z=2.1, p=0.04, or WPS-published algorithm score, z=2.23, p=0.03) (Figure 3). In contrast to vMPFC, when considering RTPJ Mentalizing>Physical activation, there was no significant correlation present in either females (r=0.19, p=0.41; r=0.16, p=0.48 when using ADOS SA+RRB score, and r=0.28, p=0.21 when using WPS-algorithm score) or males (r=0.15, p=0.55; r=0.07, p=0.77 when using ADOS SA+RRB score, and r=0.02, p=0.90 when using WPS-algorithm score), and no significant difference between the correlations (z=0.15, p=0.88; z=0.33, p=0.74 when using ADOS SA+RRB score, and z=0.96, p=0.34 when using WPS-algorithm score).

References

Baron-Cohen, S., Wheelwright, S., Robinson, J., & Woodbury-Smith, M. (2005). The Adult Asperger Assessment (AAA): a diagnostic method. *J Autism Dev Disord, 35*(6), 807-819.

Charman, T., Loth, E., Tillmann, J., Crawley, D., Wooldridge, C., Goyard, D., . . . Buitelaar, J. K. (2017). The EU-AIMS Longitudinal European Autism Project (LEAP): clinical characterisation. *Mol Autism, 8*, 27. doi:10.1186/s13229-017-0145-9

Lai, M. C., Lombardo, M. V., Pasco, G., Ruigrok, A. N., Wheelwright, S. J., Sadek, S. A., . . . Baron-Cohen, S. (2011). A behavioral comparison of male and female adults with high functioning autism spectrum conditions. *PLoS ONE, 6*(6), e20835. doi:10.1371/journal.pone.0020835

PONE-D-11-03038 [pii]

Lai, M. C., Lombardo, M. V., Ruigrok, A. N., Chakrabarti, B., Auyeung, B., Szatmari, P., . . . Consortium, M. A. (2017). Quantifying and exploring camouflaging in men and women with autism. *Autism, 21*(6), 690-702. doi:10.1177/1362361316671012

Lai, M. C., Lombardo, M. V., Ruigrok, A. N., Chakrabarti, B., Wheelwright, S. J., Auyeung, B., . . . Baron-Cohen, S. (2012). Cognition in males and females with autism: similarities and differences. *PLoS ONE, 7*(10), e47198. doi:10.1371/journal.pone.0047198

Lai, M. C., Lombardo, M. V., Suckling, J., Ruigrok, A. N., Chakrabarti, B., Ecker, C., . . . Baron-Cohen, S. (2013). Biological sex affects the neurobiology of autism. *Brain, 136*(Pt 9), 2799-2815. doi:10.1093/brain/awt216

Lombardo, M. V., Chakrabarti, B., Bullmore, E. T., & Baron-Cohen, S. (2011). Specialization of right temporo-parietal junction for mentalizing and its relation to social impairments in autism. *Neuroimage, 56*(3), 1832-1838. doi:S1053-8119(11)00223-0 [pii]

10.1016/j.neuroimage.2011.02.067

Lombardo, M. V., Chakrabarti, B., Bullmore, E. T., Sadek, S. A., Pasco, G., Wheelwright, S. J., . . . Baron-Cohen, S. (2010). Atypical neural self-representation in autism. *Brain, 133*(Pt 2), 611-624. doi:awp306 [pii]

10.1093/brain/awp306

Zeestraten, E. A., Gudbrandsen, M. C., Daly, E., de Schotten, M. T., Catani, M., Dell'Acqua, F., . . . Craig, M. C. (2017). Sex differences in frontal lobe connectivity in adults with autism spectrum conditions. *Transl Psychiatry, 7*(4), e1090. doi:10.1038/tp.2017.9
